# *Staphylococcus aureus* vertebral osteomyelitis: a single-centre retrospective cohort study with focus on oral flucloxacillin follow-up

**DOI:** 10.1007/s10096-025-05176-8

**Published:** 2025-05-28

**Authors:** Johan Wern, Bo Söderquist, Staffan Tevell

**Affiliations:** 1 Department of Infectious Diseases, Karlstad Hospital, Karlstad, Sweden; 2 School of Medical Sciences, Faculty of Medicine and Health, Örebro University, Örebro, Sweden; 3 Centre for Clinical Research and Education, Region Värmland, Karlstad, Sweden

**Keywords:** Vertebral osteomyelitis, *S. aureus* bacteraemia, Flucloxacillin, Cloxacillin, Beta-lactam antibiotics

## Abstract

**Background:**

International guidelines for *Staphylococcus aureus* vertebral osteomyelitis recommend 6 weeks of treatment, including oral follow-up using antibiotics with high bioavailability such as a fluoroquinolone/rifampicin combination. Oral flucloxacillin is not recommended due to low bioavailability and scarce evidence. However, flucloxacillin as oral follow-up treatment is common practice in Sweden based on favourable clinical experience, good tolerability, few interactions, and low ecological impact. Our aim was to review a single-centre experience of *S. aureus* vertebral osteomyelitis, with focus on flucloxacillin treatment.

**Methods:**

A single-centre retrospective cohort of patients with *Staphylococcus aureus* vertebral osteomyelitis (*n* = 40) was identified between 2010 and 2016. Patients were further stratified by antibiotic treatment strategy with focus on oral flucloxacillin therapy (*n* = 24). Primary outcomes were relapse or death within 12 months of treatment initiation, and antibiotic-related adverse effects during treatment.

**Results:**

Methicillin-susceptible *S. aureus* (MSSA) caused 38 of the infections (95%), and five patients (13%) died, all in-hospital. Flucloxacillin was used for at least 75% of the oral treatment duration in 24 patients (60%). Median antibiotic treatment duration among these patients was 125.5 days (IQR 95–182), 109 days (IQR 76–149) of which comprised oral antibiotics. There were two relapses and two deaths among the patients treated predominantly with flucloxacillin, resulting in a composite clinical cure rate of 83% (20 of 24).

**Conclusions:**

Prolonged oral flucloxacillin administration could be a potential treatment option for MSSA vertebral osteomyelitis. A prospective study of optimal treatment duration and dosing strategies for flucloxacillin in vertebral osteomyelitis is warranted.

**Supplementary Information:**

The online version contains supplementary material available at 10.1007/s10096-025-05176-8.

## Introduction

Vertebral osteomyelitis is a serious condition, with reported one-year mortality rates of 3–24% [1] and an estimated early in-hospital mortality rate of 7–14% [2]. *Staphylococcus aureus* is the most common causative pathogen, often associated with concomitant bacteraemia which in turn can be complicated by other conditions, such as infective endocarditis [3]. After an initial phase of intravenous (IV) antibiotic treatment, international guidelines recommend an oral follow-up treatment to a total (IV and oral) duration of 6 weeks, usually comprising either clindamycin, linezolid, or a fluoroquinolone (e.g., ciprofloxacin or levofloxacin) in combination with rifampicin [1, 4]. The rationale for selecting these drugs includes high oral bioavailability, good penetration into bone, and efficacy against both intracellular and biofilm-embedded staphylococci [5].

Flucloxacillin and its close relative dicloxacillin are penicillinase-resistant isoxazolyl-penicillins, active against methicillin-susceptible *S. aureus* (MSSA). Flucloxacillin is approved for intravenous treatment of MSSA infections in both the Infectious Diseases Society of America (IDSA) and French-Speaking Society of Infectious Pathology (SPILF) guidelines for vertebral osteomyelitis [1, 4]. However, due to its low and variable bioavailability, low bone penetration, high protein binding, and uncertain effect intracellularly and in biofilm [6–9], neither of these guidelines recommend it for oral follow-up treatment [1, 4]. A recent review [9] explored the current evidence for using oral flucloxacillin in osteomyelitis. Although data were scarce and primarily based on case reports and case series, the authors concluded that in contrast to the general opinion, this treatment strategy for osteomyelitis was not found to be associated with a higher rate of clinical failures. Furthermore, a short series of older studies published between 1969 and 1980, that were not included in that review, reported on PK/PD parameters and successful treatment with isoxazolyl-penicillin in a handful of cases of osteomyelitis [10–12].

Despite the limited evidence, oral follow-up treatment with flucloxacillin for bone and joint infections without implants is a common practice in Sweden. It has been recommended as first-line treatment for native vertebral osteomyelitis with or without abscesses in the Swedish Society for Infectious Diseases national guidelines [13] since the first edition was published in 2004. The recommended dosage is 1.5 g 3–4 times daily for a duration of 12(–16) weeks. Considering the short half-life of betalactam antibiotics, frequent dosing intervals are required to optimize the time above MIC target. However, achieving patient compliance using frequent dosing over long treatment durations may be challenging. Still, although the recommended treatment duration is prolonged compared to international guidelines, the Swedish national guidelines consider flucloxacillin to be an attractive and safe option regarding tolerability, interactions, and ecological impact [13, 14].

Thus, considering the limited published data, our aim was to retrospectively review a single-centre experience of *S. aureus* vertebral osteomyelitis, with focus on the safety and efficacy of flucloxacillin in the oral follow-up treatment.

## Materials and methods

This single-centre retrospective cohort study investigated patients treated for vertebral osteomyelitis at the Department of Infectious Diseases, Karlstad Central Hospital, Sweden during 2010–2016. This hospital is located in Region Värmland, which had a population of approximately 274 000 during the study period. Although Region Värmland has three hospitals in total, the 500-bed Karlstad Central Hospital is the only hospital in the region with a dedicated infectious diseases ward.

Patients treated for vertebral osteomyelitis at the Department of Infectious Diseases between 1 January 2010 and 31 December 2016 were identified through ICD-10 codes (Supplemental Table S1), and a retrospective review of medical records was performed. Patients were included if diagnosed with vertebral osteomyelitis based on clinical symptoms in combination with magnetic resonance imaging (MRI) findings at the discretion of the attending physician. If MRI was not feasible (Supplemental Table S2), findings on computed tomography (CT) or autopsy were accepted. Exclusion criteria were age < 18 years, not permanently resident in Region Värmland, initiated treatment for vertebral osteomyelitis prior to 1 January 2010, implant-associated infection with index surgery within 12 months, and isolated facet joint arthritis.

During 2010–2016, 79 patients with vertebral osteomyelitis were diagnosed, yielding a 5-year incidence of 5.8/100 000 people-years. MRI was performed in 73 (92%) patients. In four patients, where MRI was performed 1–8 days after onset of symptoms, the first MRI was negative for vertebral osteomyelitis, and diagnosis was made on a second MRI. The most common location was the lumbar region, and almost half of the infections were caused by *S. aureus* (Supplemental Table S3). In the next step, all patients with aetiologies other than *S. aureus* were excluded from the final cohort. A cut-off at ≥ 75% of the oral treatment duration was set to define flucloxacillin-based therapy. The primary outcome studied was clinical cure, defined as the absence of relapse or death at 12 months from treatment initiation. Patients who died before switching from intravenous to oral therapy (early mortality) were excluded from the final outcome analysis. Antibiotic-related side effects during treatment were studied as a secondary outcome.

## Statistics

Statistical analysis was performed using IBM SPSS Statistics (version 28.0.1.1). Data are presented as mean with standard deviation (SD) for normally distributed values, or median with interquartile range (IQR) for non-normally distributed values. An independent samples t-test (after logarithmic transformation if non-normally distributed) or Fisher’s exact test was used to assess the association between categorical variables of non-parametric data. The Mann-Whitney U non-parametric test was used for sensitivity analysis. One-sided p-values were selected for parameters expected to depart from the reference value in one direction (e.g., the presence of endocarditis is expected to lead to longer intravenous treatment duration), while all other p-values were two-sided.

## Ethics

The study protocol was approved by the Swedish Ethical Review Authority (ref: 2019-00055).

## Results

All 40 patients with vertebral osteomyelitis caused by *S. aureus* had concomitant *S. aureus* bacteraemia (SAB). The mean age was 65.1 years, and the majority (65%) were male (Table 1). Back pain and fever were the predominant symptoms (Table 1), and 16 (42%) patients presented with affected neurological status, including urine retention in 7 (19%). Diagnosis was made through MRI for 37 (93%) of the patients. For the remaining three, CT was consistent with vertebral osteomyelitis in two, and the final patient was diagnosed through histopathology at autopsy. The most common location of the vertebral osteomyelitis was the lumbar region, and seven patients had infection in at least two different levels with unaffected vertebrae in between. An abscess was present in 29 (73%) patients, among these 25 (63%) had concomitant epidural abscesses, and 13 (33%) had paravertebral abscesses (Table 2). No patients had instrumentation on the level of vertebral osteomyelitis at the onset of symptoms. One third of the patients had an additional infectious focus apart from the vertebral osteomyelitis, including infective endocarditis in five cases (13%).

**Table 1:** Baseline data, clinical symptoms, and findings for 40 patients with *S. aureus* vertebral osteomyelitis compared to the 24 patients included in the flucloxacillin cohort

	Full cohort (*n* = 40)	Flucloxacillin cohort (*n* = 24)
***Baseline data***	**n (%)**	**n (%)**
Age (mean; range; SD)	65.1 (35–87; 13.8)	66.2 (45–88; 13.0)
Male	26 (65.0)	17 (70.8)
Diabetes	10 (25.0)	6 (25.0)
Malignancy	1 (2.5)	1 (4.2)
Cirrhosis	2 (5.0)	1 (4.2)
Alcohol overconsumption	6 (15.0)	4 (16.7)
IV drug use	6 (15.0)	3 (12.5)
Haemodialysis	1 (2.5)	0 (0.0)
Central line at onset	1 (2.5)	1 (4.2)
Smoker	9 (22.5)	6 (25.0)
Immunosuppression	5 (12.5)	2 (8.3)
***Clinical symptoms and findings***	n* (%)	n* (%)
Back pain	38/39 (97.4)	24/24 (100.0)
Fever	35/40 (87.5)	20/24 (83.3)
Sepsis/septic shock	24/30 (80.0)	15/19 (78.9)
Neurological symptoms	16/38 (42.1)	11/24 (45.8)
Affected motor function	9/38 (23.7)	4/24 (16.7)
Affected sensory function	10/38 (26.3)	6/24 (25.0)
Affected reflexes	2/33 (6.1)	1/22 (4.5)
Urine retention	7/36 (19.4)	6/22 (27.2)
Positive blood culture	40/40 (100)	24/24 (100.0)
Additional infectious foci	13/39 (33.3)	5/24 (20.8)
Endocarditis^#^	5/39 (12.8)	0/24 (0.0%)
Septic arthritis	4/39 (10.2)	1/24 (4.2)
Other	9/39 (23.0)	4/24 (16.7)

^*^ missing data leading to *n* < 40 or *n* < 24

^#^ all definitive according to Duke’s criteria

**Table 2:** Localization of vertebral osteomyelitis and the presence and location of abscesses

**Full cohort (** ***n*** ** = 40)**	***n*** **(%)**	**Paravertebral abscess** ***n*** **(%)**	**Epidural abscess** ***n*** **(%)**	**Iliopsoas abscess** ***n*** **(%)**
Cervical	5 (12.5)	2 (15.4)	5 (20.0)	0 (0.0)
Cervical + lumbar	2 (5.0)	2 (15.4)	1 (4.0)	1 (16.7)
Cervical + lumbar/sacral	1 (2.5)	1 (7.7)	1 (4.0)	0 (0.0)
Cervical + thoracic + lumbar	1 (2.5)	0 (0.0)	1 (4.0)	1 (16.7)
Thoracic	5 (12.5)	2 (15.4)	2 (8.0)	0 (0.0)
Thoracic + lumbar	3 (7.5)	2 (15.4)	2 (8.0)	0 (0.0)
Thoracic + lumbar/sacral	1 (2.5)	1 (7.7)	1 (4.0)	1 (16.7)
Lumbar	17 (42.5)	1 (7.7)	8 (32.0)	3 (50.0)
Lumbar + sacral	5 (5.0)	2 (15.4)	4 (16.0)	0 (0.0)
Total	40 (100)	13 (100)	25 (100)	6 (100)
**Flucloxacillin cohort (** ***n*** ** = 24)**	**n (%)**	**Paravertebral abscess n (%)**	**Epidural abscess n (%)**	**Iliopsoas abscess ** **n (%)**
Cervical	2 (8.3)	1 (12.5)	2 (12.5)	0 (0.0)
Cervical + lumbar	2 (8.3)	2 (25.0)	1 (6.3)	1 (0.25)
Cervical + lumbar/sacral	0 (0.0)	0 (0.0)	0 (0.0)	0 (0.0)
Cervical + thoracic + lumbar	1 (4.2)	0 (0.0)	1 (6.3)	1 (0.25)
Thoracic	1 (4.2)	0 (0.0)	1 (6.3)	0 (0.0)
Thoracic + lumbar	2 (8.3)	2 (25.0)	2 (12.5)	0 (0.0)
Thoracic + lumbar/sacral	0 (0.0)	0 (0.0)	0 (0.0)	0 (0.0)
Lumbar	12 (50.0)	1 (12.5)	5 (31.3)	2 (0.5)
Lumbar + sacral	4 (16.7)	2 (25.0)	4 (25.0)	0 (0.0)
Total	24 (100.0)	8 (100.0)	16 (100.0)	4 (100.0)

The *S. aureus* isolates were methicillin susceptible in 38/40 (95%) cases. Empirical treatment was started in 31 (78%) of the patients, and no patient received empirical vancomycin. The most common initial IV antibiotic was cefotaxime (*n* = 16; 40%), followed by cloxacillin (*n* = 14; 35%). IV treatment was administered for a median of 20.5 (range: 4–173) days, but there was a statistically significant difference when stratified on the absence versus presence of additional infectious foci such as infective endocarditis, septic arthritis or non-spinal abscesses (19.5 vs. 29.0 days, *p* = 0.02). However, when combining the IV and oral treatment, the median duration was 123 (range: 4–257) days, with no difference between these groups (111 vs. 128 days, *p* = 0.74). There was still no difference in median treatment duration when excluding patients who died during treatment; 116 (range: 52–249) vs. 132 (range: 42–257) days (*p = 0.38*) (Table 3). Most patients (*n* = 35, 87.5%) received cloxacillin at some point during the IV treatment, and 24 (60.0%) received intravenous cloxacillin for at least 75% of the IV treatment duration.

**Table 3:** Antibiotic treatment duration of *S. aureus* vertebral osteomyelitis. One of the 40 patients died shortly after admission and was not assessed for other infectious foci. In five patients, no oral treatment was given. T-test comparing treatment durations depending on the presence or absence of additional infectious foci

	Full cohort	Flucloxacillin cohort	Vertebral osteomyelitis only	Vertebral osteomyelitis and additional foci	*p*-value
	Median (IQR)	Median (IQR)	Median (IQR)	Median (IQR)	
In-hospital time	32.5 (25–56)	34 (26–56)	32.5 (24–57)	40 (26.5–58)	0.33*
IV treatment, days	20.5 (16-32.5)	19.5 (16–31)	19.5 (15–31)	29 (18–40)	0.02*
Oral treatment, days	99 (75–149)	109 (76–149)	84 (69.5–137)	91 (12.5-156.5)	0.92
Total treatment duration	123 (93–181)	125.5 (95–182)	111 (92-158.5)	128 (66.5-188.5)	0.74
Total treatment duration#	128.5 (97–182)	135 (101–185)	116 (96–162)	132 (118–189)	0.38

^#^ excluding patients who died during treatment

^*^ one-sided t-test

Clinical cure was achieved in 82% (33 of 40) in the entire cohort. Five patients (12%) died, all during their hospital stay, and there was a significant difference in age between the deceased and surviving patients (mean, SD: 81.2 ± 3,3 vs. 62.9 ± 13.2, *p* < 0.001). Epidural abscesses were diagnosed in 25 patients, four of whom died during the intravenous phase of treatment. Seven patients underwent spinal surgery, whereof five implant surgeries. Both patients that underwent surgical source control without spinal implants died in-hospital.

Only 34 patients were evaluable regarding oral treatment, as five of the original 40 received intravenous antibiotics only (four who died during the intravenous treatment phase and one with an MRSA infection who completed the full course intravenously) while one was lost to follow-up. Among these 34, oral flucloxacillin was used in 32 patients (94%), whereof 24 (70%) received flucloxacillin for ≥ 75% of the oral treatment duration, per the pre-defined cutoff. Among the remaining patients, clindamycin (*n* = 8) was the most commonly prescribed antibiotic. None of the patients undergoing spinal implant surgery received prolonged oral flucloxacillin follow-up however two patients received ciprofloxacin/rifampicin combination therapy.

**Fig. 1:**
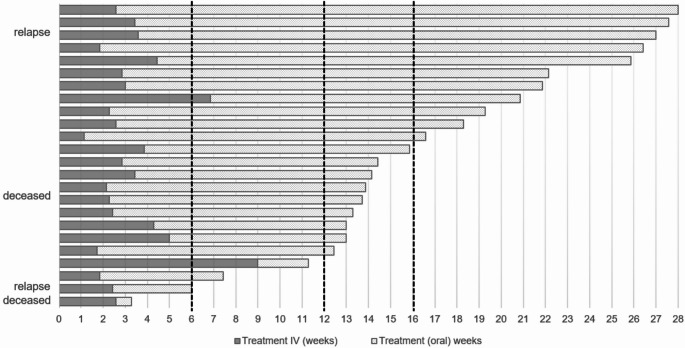
Treatment duration in the oral flucloxacillin group. The first line marks the 6 weeks recommended in international guidelines, the second and third line marks the 12–16 weeks recommended in Swedish guidelines

The median IV treatment duration in the flucloxacillin cohort was 19.5 (IQR 16–31) days (Table 3). Further analysis of this cohort revealed that all these patients received flucloxacillin throughout the treatment period (100%). The treatment duration was longer than recommended in the Swedish national guidelines, as 11 of 24 patients (46%) received more than 16 weeks of antibiotics (Fig. 1). Furthermore, six patients (25%) received concomitant fusidic acid for at least 1 week, whereof two for the entire treatment duration.

In the oral flucloxacillin cohort, two patients died in-hospital during treatment (1–2 weeks after oral switch), and two relapses occurred early after discontinuation of antibiotic treatment (six and 21 days, respectively). Thus, 91% (20/22) of the patients were considered cured among those who completed their flucloxacillin-based oral treatment, while the clinical cure rate for the composite endpoint of death and relapse was 83% (20 of 24).

Adverse events related to antibiotics were documented in five patients. Three patients treated with cloxacillin or flucloxacillin experienced severe adverse events, manifested by vasculitis (*n* = 1), nephrotoxicity (*n* = 1), and hepatotoxicity (*n* = 1). These adverse events were reversible after switching to clindamycin, and none died or experienced relapse. In addition, two patients experienced nausea, one on flucloxacillin and one on ciprofloxacin/rifampicin. No patients experienced *Clostridioides difficile* infection.

## Discussion

In this study, we aimed to review a single-centre experience of *S. aureus* vertebral osteomyelitis and assess the efficacy and safety of flucloxacillin as oral follow-up treatment. Among 24 patients treated with flucloxacillin as oral follow-up, the clinical cure rate at 12 months was 83%, indicating that prolonged flucloxacillin treatment might achieve cure rates similar to those reported by Park et al. [15] and Bernard et al. [16]. Thus, in selected cases, flucloxacillin could be a potential option for oral follow-up in these infections.

Only scarce data are available on the outcome after oral flucloxacillin follow-up treatment for vertebral osteomyelitis. No relapses were detected in a clinical and pharmacokinetic evaluation of a combination of flucloxacillin and probenecid in 1980 [12]; however, only four patients with osteomyelitis were included in the investigation, and the published article did not contain any extractable patient details. Beronius et al. [17] presented a Swedish cohort consisting of 42 patients, of whom 24 (57%) had received flucloxacillin as oral treatment. In general, the intravenous treatment duration was shorter compared to the present study, whereas the oral follow-up was longer (median 10 days and 179 days, respectively). The only relapse detected was in a patient receiving seven months of dicloxacillin as follow-up. Two additional case reports (without relapses) were presented in a recent narrative review [9].

As cephalosporins share mechanisms of action with other beta-lactams, their effectiveness in intracellular or biofilm environments is not expected to differ. The bioavailability of oral cephalosporins is variable, and the group includes drugs with high bioavailability (e.g., 95% for cephalexin). Okunmura et al. reported an 87% success rate (*n* = 15) for oral follow-up with cephalexin after at least 3 weeks of intravenous treatment, with a median total treatment duration of 86 days [18]. In another retrospective cohort study, a successful outcome was reported in 86% (*n* = 29) of patients treated with intravenous beta-lactam antibiotics followed by oral first-generation cephalosporins or amoxicillin/clavulanate for a median duration of 55–80 days [19]. There was no significant difference when comparing this oral step-down to patients receiving intravenous beta-lactam antibiotics for the entire treatment duration. Furthermore, longer treatments were associated with relapse, indicating a selection bias where the more complex high-risk cases were treated longer. In the present study, outcomes were similar, but the treatment durations exceeded those recommended by the Swedish national guidelines. As one of the relapses occurred in one of the patients receiving more than six months of treatment while most patients receiving 12–16 weeks of treatment achieved clinical cure, it is possible that a similar selection bias impacts the treatment duration in the present study.

Bernard et al. [16] demonstrated that 6 weeks of antibiotic treatment was non-inferior to 12 weeks in vertebral osteomyelitis. However, the majority of patients received antibiotic combinations, mostly fluoroquinolone/rifampicin combinations (44%). Park et al. [15] identified methicillin-resistant *S. aureus*, undrained paravertebral/psoas abscesses, and end-stage renal disease as risk factors for relapse and recommended prolonged treatment (> 8 weeks) to these patients. It is notable that the studied populations were different, as the cohort studied by Bernard et al. included 19% abscesses, compared to 49% in the study by Park et al. [20]. In the present cohort, 72.5% of patients had an abscess, including 40% with paravertebral and/or psoas abscesses. One patient who relapsed after 6 weeks of treatment (including 4 weeks of flucloxacillin) had epidural and psoas abscesses, and was thus high-risk for relapse according to Park et al. Nevertheless, the optimal treatment duration for vertebral osteomyelitis when using flucloxacillin is unknown. In this cohort, the median treatment duration was 16.9 weeks, but five patients were treated for ≤ 13 weeks without relapse, even in the presence of abscesses. However, the above-cited data from oral cephalosporins [18, 19] suggest that 12 weeks could be sufficient for oral betalactam follow up treatment. However, considering the need for source control as well as the conditions for adequate antibiotic exposure is important when planning for oral follow-up.

The bioavailability and protein binding ability of flucloxacillin have led to concerns regarding its efficacy in complicated infections such as vertebral osteomyelitis. In a sub-study of the POET trial on oral treatment for infective endocarditis [21], the probability of target attainment (PTA) for the comparable oral drug dicloxacillin was only 9–17%. However, these calculations may represent an underestimation. First, the uptake of oral flucloxacillin is variable. Dijkmans et al. [22] demonstrated insufficient drug absorption in 13% of patients and proposed an oral absorption test at the time of switching from intravenous to oral treatment. Second, the high protein binding (97% for dicloxacillin, 95% for flucloxacillin) implies a low free fraction to mediate the antibiotic effect. However, estimating the unbound concentration using a fixed protein binding factor may underestimate unbound concentrations, especially in critically ill patients [23–25]. Most likely, this phenomenon is caused by hypoalbuminaemia leading to higher free fractions, which is not only present in patients requiring critical care, but also common among patients with severe bacterial infections [24]. Finally, recent animal data [26] suggest a relatively low PK/PD target (20%*f*T > 0.25xMIC for a 2-log reduction of MSSA), implying that previously calculated PTAs for flucloxacillin in MSSA infections might be too low [26].

Another strategy for better PTA is to prolong the half-life of flucloxacillin through interference with tubular secretion by adding probenecid to the treatment [12]. Up to 5.5-fold improvements of *f*T > MIC have been demonstrated through alteration of the concentration-time curve shape by combining these drugs. This strategy could provide better conditions to achieve patient compliance through longer dosing intervals with unaltered PTA [27].

Thus, there are several uncertainties in the assumptions used when calculating a low PTA for flucloxacillin in the treatment of MSSA infections, and there are also measures to be taken to optimize treatment conditions. Therapeutic drug monitoring (TDM) was not performed on the patients included in this study; however, one of the two patients who relapsed had a high body weight, and hence might have been underdosed. It is possible that a structured algorithm utilizing an oral absorption test and TDM might aid in the selection of suitable patients and optimizing dosing strategies if oral flucloxacillin follow-up treatment is considered.

Another concern in flucloxacillin use is the potential adverse effects, especially idiosyncratic hepatotoxicity [28]. In this cohort, the frequency of documented side-effects requiring cessation of oral flucloxacillin was 6%, including 1 patient (3%) with hepatotoxicity. All side-effects were reversible after switching drug. It has been estimated that flucloxacillin-associated liver damage will occur in 1 in 7 000 patients in a general population, but the risk varies with age, being 15-fold higher in patients aged over 70 years compared to patients under 50 years [29]. Female sex, HLA-B*57:01 genotype, and pre-existing kidney stones have also been associated with elevated risk for hepatotoxicity during flucloxacillin treatment [30]. In comparison, the incidence of hepatotoxicity has been reported to be 3% for fluoroquinolone treatment [31] and 1% for rifampicin monotherapy [32]. In a prospective study on treatment of diabetic foot osteomyelitis, 40% of patients experienced adverse events, most commonly gastrointestinal which were all attributable to rifampicin. Induction of metabolic enzymes (e.g., CYP3A4) leading to extensive drug-drug interactions further complicates rifampicin usage [32]. Another serious adverse event after antibiotic treatment is *Clostridioides difficile* infection. Interestingly, the adjusted odds ratio (aOR) for dicloxacillin is similar to that of ciprofloxacin, despite being a more narrow-spectrum antibiotic. In that regard, levofloxacin seems to be a better option among fluoroquinolones, while clindamycin, recommended as second-line therapy by IDSA and SPILF, was associated with the highest aOR of all studied antibiotics in a matched case-control study [1, 4, 33]. Thus, all potential treatment options for vertebral osteomyelitis have their pros and cons, and it is crucial to be aware of these risk factors when selecting the therapy least likely to cause harm to the individual patient.

Limitations of this study include its retrospective nature, including the possibility that cases were not found due to coding errors, the small number of patients, and the low occurrence of relapses. From this cohort, it is not possible to draw conclusions regarding the optimal treatment duration. Moreover, a larger cohort would provide a better estimate of the safety and efficacy of flucloxacillin treatment in vertebral osteomyelitis.

In conclusion, prolonged flucloxacillin administration as an oral follow-up for MSSA vertebral osteomyelitis resulted in an 83% (20 of 24) clinical cure rate in this small retrospective cohort. The optimal treatment duration is unknown, but 12 (–16) weeks may be sufficient even in the presence of abscesses, provided that adequate drug absorption is ensured. A prospective study on treatment duration utilising TDM to optimize conditions for target attainment might shed further light on the optimal usage of flucloxacillin in the setting of MSSA bacteraemia complicated by vertebral osteomyelitis.

## Electronic supplementary material

Below is the link to the electronic supplementary material.

Supplementary Material 1

## Data Availability

The datasets generated during and/or analysed during the current study are not publicly available due to reasons of sensitivity but are available from the corresponding author on reasonable request.

## Declarations

### Transparency declaration

B.S. has been a member of an advisory board at ADVANZ PHARMA, and has also received speaker’s fees from Correvio Pharma Corp and ADVANZ PHARMA. All other authors: none to declare.
