# Supplementary material for: Staphylococcus aureus vertebral osteomyelitis: a single-centre retrospective cohort study with focus on oral flucloxacillin follow-up
Source: Eur J Clin Microbiol Infect Dis. 2025 May 28;44(9):2077–84. doi: 10.1007/s10096-025-05176-8 (PMC12457446; doi:10.1007/s10096-025-05176-8)
Supplement: Supplementary file 1 — Supplementary Material 1 [file 10096_2025_5176_MOESM1_ESM.docx]

# Supplementary information

# Flucloxacillin treatment for *Staphylococcus aureus* vertebral osteomyelitis: a single-centre retrospective cohort study

Johan Wern, Bo Söderquist, Staffan Tevell

## Supplemental Table S1

ICD-10 codes used for identification of patients with vertebral osteomyelitis.

| A02.2, A18.0, B67.2, M46*, M49.0, M49.1, M49.2, M49.3, M90.0 |
| --- |

## Supplemental Table S2

Reasons for not performing MRI

| Pacemaker (n=2) |
| --- |
| Metal clips in CNS (n=1) |
| Too large for MRI (n=1) |
| Acute surgery after CT (n=1) |
| Poor prognosis regardless of results of MRI (n=1) |

## Supplemental Table S3

Causative pathogens in all vertebral osteomyelitis at Central Hospital, Karlstad, 2010–2016.

| **Pathogen** | **n(%)** |
| --- | --- |
| *Staphylococcus aureus^*^* | 40 (46.5) |
| *Streptococcus spp.* | 14 (16.3) |
| *Mycobacterium tuberculosis* | 6 (7.0) |
| *Enterococcus faecalis* | 2 (2.3) |
| *Enterobacterales* | 4 (4.7) |
| Polymicrobial | 4 (4.7) |
| Other | 6 (7.0) |
| Culture negative | 10 (11.6) |
| Total | 86 (100) |
| ^*^Two (5%) were MRSA |  |
